# The Role of the Western Diet and Oral Microbiota in Parkinson’s Disease

**DOI:** 10.3390/nu14020355

**Published:** 2022-01-14

**Authors:** Barbara Zapała, Tomasz Stefura, Tomasz Milewicz, Julia Wątor, Monika Piwowar, Magdalena Wójcik-Pędziwiatr, Magdalena Doręgowska, Alicja Dudek, Zuzanna Jania, Monika Rudzińska-Bar

**Affiliations:** 1 Department of Clinical Biochemistry, Jagiellonian University Medical College, 31-066 Krakow, Poland; Zuzannajania@gmail.com; 2 2nd Department of General Surgery, Jagiellonian University Medical College, 31-008 Krakow, Poland; tomasz.stefura@gmail.com (T.S.); ala.ddudek@gmail.com (A.D.); 3 Department of Gynaecological Endocrinology and Gynaecology, Jagiellonian University Medical College, 31-501 Krakow, Poland; tomasz.milewicz@uj.edu.pl; 4 Faculty of Medical Sciences in Zabrze, Medical University of Silesia, 41-800 Katowice, Poland; julia.katarzyna.wator@gmail.com; 5 Department of Bioinformatics and Telemedicine, Faculty of Medicine, Jagiellonian University Medical College, 31-034 Krakow, Poland; monika.piwowar@uj.edu.pl; 6 Department of Neurology, Andrzej Frycz Modrzewski Krakow University, 30-705 Krakow, Poland; m.pedziwiatr@szpitaljp2.krakow.pl (M.W.-P.); mdoregowska@afm.edu.pl (M.D.); mrudzinska@afm.edu.pl (M.R.-B.)

**Keywords:** Parkinson’s disease, the Western diet, oral microbiota

## Abstract

The type of diet not only affects the composition of the oral microflora but is also one of the more critical factors associated with an increased risk of Parkinson’s disease, PD. This study compared diet preferences and oral microbiota profiles in patients with PD vs. healthy controls. This study compared the oral microbiota composition of 59 patients with PD and 108 healthy controls (without neurodegeneration) using 16S rRNA gene amplicon sequencing. According to results, oral microbiota in patients with PD is different compared from healthy controls. In particular, decreased abundance of Proteobacteria, Pastescibacteria, and Tenercutes was observed. The oral cavity of patients with PD was characterized by the high relative abundance of bacteria from the genera *Prevotella*, *Streptococcus*, and *Lactobaccillus.* There were also differences in food preferences between patients with PD and healthy controls, which revealed significantly higher intake of margarine, fish, red meat, cereals products, avocado, and olives in the patients with PD relative to healthy controls. Strong positive and negative correlations between specific food products and microbial taxa were identified.

## 1. Introduction

PD is the second most common neurodegenerative disorder, affecting up to 1% of the population above 60 and 4–5% of those above 80 [1,2]. Men are about 1.5 times more likely than women to develop PD among the patients. Due to the acceleration of the aging of Western populations, incidence above the current 1-in-800 is predicted [3]. When considering that less than 10% of PD is associated with specific genetic changes, researchers are still looking for environmental risk factors causing the disease. Indeed, findings regarding the role of dietary factors in PD are conflicting. There is some evidence for a protective role of caffeine and a deleterious role of dairy products. Recently, the MIND diet, which is based on the Mediterranean and DASH diets, has also been associated with a delayed onset of PD [4,5,6]. The most popular in Europe, the Western diet, is characterized by high caloric intake of energy-dense foods, high in saturated and omega-6 (ω6) fatty acids, refined sugars, excessive salt intake, and low consumption of omega-3 (ω3) fatty acids and fiber. This diet, mainly enriched in high quantities of animal saturated fats, canned fruits and vegetables, soda, fried foods, beef, ice cream, and cheese, has been widely associated with an increased risk of developing PD [7,8,9,10]. On the other side, the Mediterranean diet correlates with decreased risk of developing PD and its components, such as fresh vegetables and fruit, nuts, seeds, fish, olive oil, wine, fresh herbs, and spices. Furthermore, consumption of flavonoid-rich foods (e.g., tea, berry fruits, apples, red wine, and orange juice) seem to be associated with a lower risk of developing PD.

Polyunsaturated fatty acids (PUFA) were inversely correlated with PD development [11,12,13,14]. Additionally, several reports have even shown that higher consumption of ω3 fatty acids is associated with a lower risk of PD development, and this has provoked suspicion that ω3 fatty acids influence brain function [11,12,13,14]. There are multiple mechanisms by which a diet can impact the body’s homeostasis, for example, by the direct influence of dietary components as vitamins or fats. However, the most intriguing point now is the indirect influence of diet components on the human microbiome [13].

The intestinal microbiota was significantly different in patients with PD compared to healthy controls [15,16,17]. However, it is worth emphasizing that gut microbiota is disrupted among patients diagnosed with PD, and disruptions in oral and even nasal microbiota profiles may influence the development and progression of the disease. Throughout life, many factors influence oral microbiota, including environmental factors, dietary habits, host genetics, hygiene practice, and medications. Although oral microbial communities are highly variable, prior evidence has demonstrated that microbiome perturbations in the oral cavity can influence health and provoke disease [15,16,17]. For example, it has been reported that opportunistic pathogens can provoke diseases such as pneumonia, and such pathogens were observed to be more abundant in the mouths of people with PD than in healthy individuals. Thus, changes in oral cavity bacteria in patients with PD could influence disease symptoms affecting the mouth, particularly drooling and difficulty swallowing, which are well-known symptoms of PD [15,16,17]. The opportunistic pathogens may be hazardous under certain circumstances. For example, in PD, oral health is poorer than in healthy individuals [18]. PD patients have difficulties flossing or brushing the teeth, which, when combined with increased drooling and difficulty swallowing, provoke plaque building in the mouth and cause the formation of a fertile breeding ground for bacteria [18]. It was demonstrated that Streptococcus pneumoniae was an opportunistic pathogen responsible for provoking pneumonia. In PD patients, symptoms such as aspiration pneumonia, caused by inhaling food, drinks, vomits, or saliva into the lungs, are a very common cause of death [18]. Moreover, it has been observed that PD patients with trouble swallowing and excess drooling were characterized by a significantly higher abundance of *S. pneumoniae* in the oral cavity. What was very important in the study reported by Rozas et al. was that there were no statistically significant differences observed in oral hygiene habits between the patients with PD and healthy controls [18]. These results strongly suggested that poor oral health observed in PD patients is not caused by the reduced frequency or efficiency of oral hygiene but rather is linked to disease-specific factors resulting in increased abundance of certain bacteria, which can initiate an oral disease [18]. There are much fewer reports describing oral microbiota concerning PD. However, it has been shown that there are differences in oral microbiome profiles in patients with PD compared to healthy controls. Previous studies report that oral microbiota in patients with PD is much more enriched in opportunistic pathogens when compared to healthy controls [15,16,17].

This study compared diet preferences and oral microbiota profiles in patients with PD vs. healthy controls.

## 2. Materials and Methods

We performed a cross-sectional, descriptive, and comparative study including 59 patients with PD and 108 healthy controls (without neurodegeneration), ranging from 51 to 82 years old. Patients with PD were recruited from one of the hospital centers in Krakow, Poland, and healthy controls were volunteers. In the group of patients with PD, the stage of disease progression was assessed using Hoehn and Yahr staging (H & Y) and Movement Disorder Society Unified Parkinson’s Disease Rating Scale part III (MDS-UPDRS Part III) score during ON time. The patients with PD were excluded from this study when diagnosed with other neurological diseases such as systemic or neurologic infections, inflammatory or autoimmune diseases, atypical parkinsonism syndrome, and vascular parkinsonism. The exclusion criterium in that group also was brain surgery, concomitant psychiatric diseases such as schizophrenia or bipolar disorder, preliminary baseline evaluations. Patients with confirmed or suspected gastrointestinal malignant tumors or other gastrointestinal diseases were excluded. Patients with PD with a medication history of using antibiotics or probiotics over the last three months or therapy based upon steroids, non-steroidal anti-inflammatory drugs, or history of gastrointestinal surgery (e.g., gastro-resection or major intestinal surgery) did not participate in this study. Smokers were also excluded from the study. Participants with complete dentures or removable dental prostheses, or the presence of fewer than 16 natural teeth, were excluded from the study. The cognitive and functional status were scored using the Mini-Mental State Examination (MMSE) and the Montreal Cognitive Assessment (MoCA).

Additionally, computed tomography scanning or magnetic resonance imaging was performed in PD patients to exclude vascular parkinsonism. The detailed demographic data and medical history were collected using a set of questionnaires. The healthy volunteers were matched for age and were enrolled in the study based on exclusion criteria: any medical history for neurological, immunological, gastrointestinal diseases, and smoking.

All participants received a questionnaire to analyze their food preferences. The questionnaire consisted of two parts and was performed before sampling. The first part was used to determine anthropometric parameters such as body weight, height, age, gender, socioeconomic, educational status, and medical history. The second section included 33 foods, including ingredients typical of the Western and Mediterranean diets. The second part also included questions about preparing food such as boiling, steaming, baking, grilling, and frying. Food items were listed under significant food groups such as vegetables, fruit, cereals, legumes, milk, dairy products, meat and fish, eggs, oils, fats, drinks (including alcohol drinks, coffee, and tea), and snacks or fast food. Respondents reported the frequency of consumption of each food product as always, above three times per day, twice a day, once a day, 4–6 times per week, 2–3 a week, once a week, monthly, or never.

Oral samples were collected with BactiSwab™ NPG Collection and Transport System (ThermoFisher Scientific, Waltham, MA, USA), which has a unique cap design to reduce contamination during sample collection and during the DNA isolation stage, which was critical for this microbiome studies. The inner surfaces of the buccal mucosa, tongue, and hard palate were rubbed with the swab several times for at least 10 s, then the swab samples were transferred to the tubes and immediately stored at −80 °C. Before oral sampling, all participants were well informed about refraining from eating or drinking for at least 30 min before the sample collection. The commercially available QIAamp BiOstic Bacteremia DNA Kit (QIAGEN, Hilden, Germany) extracted bacterial genomic DNA. The bacterial genomic DNA was extracted from buccal swabs. During the extraction, all steps followed the manufacturer’s protocol. The extracted DNA was estimated quantitatively and qualitatively using spectrophotometer NanoDrop ND-1000 (Thermo Electron Corporation, West Palm Beach, FL, USA) and fluorometer Qubit 4 (Invitrogen, Waltham, MA, USA). Then, all isolates were stored at −20 °C until further analysis. Blood samples were collected from all study participants to measure biochemical parameters such as triglyceride, total cholesterol, high-density lipoprotein (HDL), low-density lipoprotein (LDL), alanine aminotransferase (ALT), aspartate aminotransferase (AST), white blood cells (WBCs), and red blood cells (RBCs) platelets (PLT).

A sequencing library of the 16S rRNA gene V3 and V4 regions was constructed using gene-specific primers adapted from the Klindworth et al. publication [19]. The libraries were prepared under the protocol for Preparing 16S Ribosomal RNA Gene Amplicons for the Illumina MiSeq System. The PCR-based amplification was performed following the protocol of the manufacturers of the KAPA HiFi HotStart ReadyMix (ROCHE, Basel, Switzerland). Amplification was performed under the following thermocycling: 95 °C for 1 min, 55 °C for 1 min, then 72 °C for 1 min for 30 cycles, and a final extension step at 72 °C for 5 min. The PCR products were then indexed with specific sequencing adapters using Nextera XT Index Kit v2 from Illumina. The indexing step was performed in a thermocycler using the following steps: 95 °C for 3 min, eight cycles of 95 °C for 30 s, 55 °C for 30 s, 72 °C for 30 s, and a final extension at 72 °C for 5 min and hold at 4 °C. The sequencing was performed on the MiSeq instrument (Illumina, San Diego, CA, USA) using a 300 × 2 V3 Kit and PhiX Control V3 from Illumina. The Qubit 4.0 Fluorometer (Invitrogen, Waltham, MA, USA) and Bioanalyzer (Agilent, Santa Clara, CA, USA) were used to assess the integrity and size (~630 bp) of amplicons. Prior to sequencing, the amplicons were pooled in equimolar concentrations. The raw reads of 16S rRNA gene sequences generated as FASTQ formats were filtered using the Illumina16S Metagenomics workflow to obtain high-quality reads. Then, the high-quality sequences were clustered into operational taxonomic units (OTUs) at 99.9% identity based on the Greengenes Database and the algorithm with the high-performance implementation of the Ribosomal Database Project (RDP) classifier, which was described by Wang Q. et al. in 2007 [20]. QIIME 2.0 software with Python scripts was applied to calculate alpha- and beta-diversity [21]. Alpha-diversity was measured based on the sequence similarity at 97% level. The number of unique OTUs found in each sample was assessed to present the richness. The results were presented as ACE and Chao1 indices. The Shannon, Simpson, and Fisher estimators measured both the richness and evenness within individual samples and both experimental groups [21,22]. Beta-diversity, calculated as the distance and dissimilarities between microbial communities, was determined using Jaccard, Bray–Curtis, and Jensen–Shannon Divergence indices. These results were visualized by principal coordinate analysis (PCoA) [23]. The statistically significant differences based on beta-diversity of the whole microbiome structure between patients with PD and healthy controls were calculated using a per-mutational multivariate analysis of variance (PERMANOVA). LEfSe and MicrobiomeAnalyst were then applied to perform the clustering and statistical analysis [24,25]. The linear discriminant analysis (LDA) effect size from LEfSe was adapted to indicate the most statistically significant features of oral microbiota. The discovered microbial biomarkers with statistical significance and biological relevance were de-scribed based on the normalized relative abundance matrix, the Kruskal–Wallis rank-sum test, the significant alpha at 0.05, and the effect size threshold of 2. The median abundance and the non-parametric Wilcoxon Rank Sum test were used to discover statistically significant taxonomic differences between microbial communities and abundance profiles of two experimental groups [26]. Raw data of the food preferences from questionnaires were statistically analyzed with the statistical environment R version 3.6.0, PSPP software, and MS Office 2019. The descriptive statistics were prepared, and frequency distribution was calculated and presented as means and standard deviations (SD). Adjustments were applied for age, sex, BMI, and kilocalories. Multivariate analysis was performed to determine the statistically significant differences of the means when comparing both groups’ results. The Chi-square and Fisher’s tests were used to analyze the variables on the nominal scale. The Student *t*-test t, the Mann–Whitney U, and the Kruskal–Wallis test were adapted for the quantitative variables. A *p*-value of <0.05 was considered significant.

The study was performed following the highest ethical standards of the hospital (in which the patients were recruited), national guidelines, and the Helsinki Declaration. All protocols for the study were approved by the Ethics Committee of Jagiellonian University Medical College (approvement number 1072.6120.267.2019) and written informed consent was obtained from each subject before enrollment.

## 3. Results

### 3.1. Participants

Patients with PD ranged from 55 to 82 years, whereas the healthy controls ranged from 51 to 82 years. The average age of patients with PD was 69 ± 7 years, and 24 (41%) were female. The healthy controls’ average age was 64 ± 7 years, and 63 (58%) were female. In the group of patients with PD, the mean BMI was 26.3 ± 3 kg/m^2^, and in the control group, it was 25.8 ± 4 kg/m^2^, which fall to within the overweight range but not obese. The average duration of the disease totaled 7.32 years (standard deviation 6.67). In the analysis, patients with PD included in the research group showed a grade of 2.04 on the five-point Hoehn and Yahr scale (standard deviation 0.71). The Mini-Mental State Examination scored 26.33 (standard deviation 1.51), and the Montreal Cognitive Assessment scale scored 21.8 (5.15). Both indicators denoted mild cognitive impairment among patients with PD. No significant differences in the analyzed clinical parameters and anthropometric characteristics were observed between patients with PD and healthy controls (Table 1 and Table 2).

### 3.2. Differences in Diet Preferences between Patients with PD and Healthy Controls

**Figure 1:**
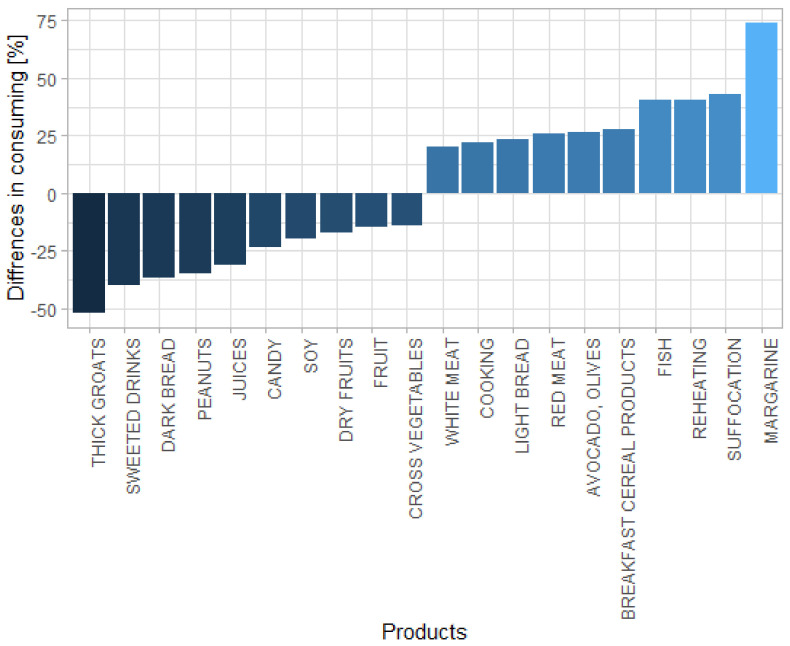
Differences in food preferences between patients with PD and the healthy controls.

Patients with PD consumed more margarine (*p* < 0.007), breakfast cereal products (*p* < 0.030), and avocado and olives (*p* < 0.001). In patients with PD, the fish intake was greater than in the controls (*p* < 0.001). However, the patients with PD more frequently consumed red meat than did the healthy controls (*p* < 0.015) (Figure 1 and Table 3). Lower consumption of thick groats (*p* < 0.001), dark bread (*p* < 0.001), peanuts (*p* < 0.001), juices (*p* < 0.004), soy (*p* < 0.028), fresh fruits (*p* < 0.008), and cross vegetables (*p* < 0.001) was observed in patients with PD when compared to the healthy controls (Figure 1 and Table 4). Sweetened drinks intake was lower in patients with PD (*p* < 0.012) compared with the healthy controls.

There were noticed statistical differences only in vitamin C consumption between the patients with PD and the healthy controls. The patients with PD consumed lower doses of vitamin C when compared to the healthy controls (*p* < 0.01). There were no statistical differences between groups concerning vitamin A (*p* < 0.54), vitamin E, (*p* < 0.67), and folic acid (*p* < 0.14). The consumption of high animal fat was higher in patients with PD (*p* < 0.02). There were also statistically significant differences in animal milk intake between the patients with PD and the healthy controls (*p* < 0.00). The patients with PD consumed much fewer carbohydrates than the normal range predicted. The diet of PD patients was also rich in proteins, and they consumed more proteins than was recommended. Moreover, we noticed that the diet of PD patients was a low-fat diet. The average age in the control group was 64.21 ± 10.23 years; the characteristics of the PD and the healthy controls are shown in Table 1.

### 3.3. Microbiota Composition in Patients with PD Group vs. Healthy Controls

We obtained 3,329,002 reads of the 16S RNA genes. The mean number of reads per sample was 47,557 (367–102,569). The total number of OTU identified was 346.

**Figure 2:**
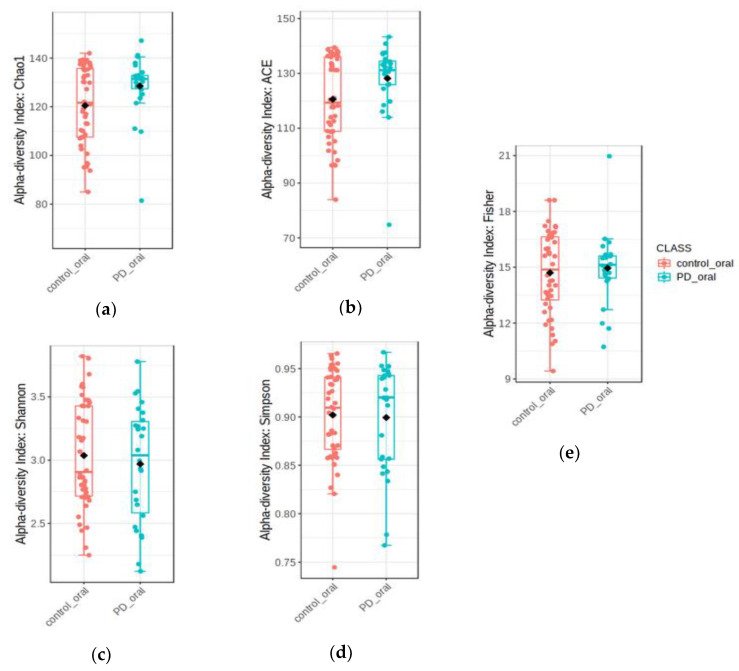
Comparisons of different alpha-diversity indices in patients with PD (PDG) and healthy controls (CG). (**a**) Chao1, *p*-value: *p* < 0.02 between PDG and CS. (**b**) ACE *p* < 0.03 between PDG and CG. (**c**) Shannon diversity index (*p* < 0.55) among patients with PD with different severity. (**d**) Simpson diversity index (*p* < 0.84) among patients with PD with different severity. (**e**) Fisher diversity index (*p* < 0.62) among patients with PD with different severity.

Alpha-diversity of analyzed samples is presented in Figure 2. Microbiota from the control group revealed significantly higher species richness than the microbiota of patients with PD (Chao1 index *p* < 0.02 ACE index *p* < 0.03). The specific weight of microbiota in the healthy controls is more diverse than in patients with PD.

**Figure 3:**
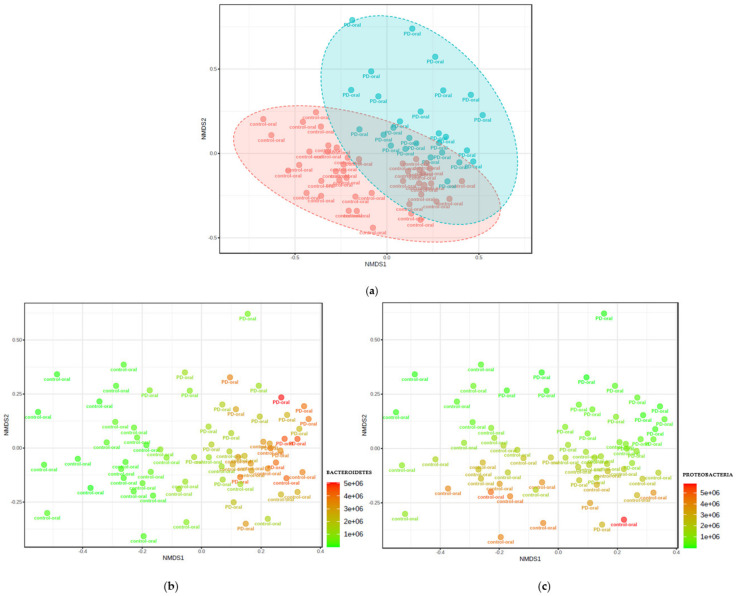
Visualization of oral microbial communities in patients with PD and healthy controls using non-metric multidimensional scheme. D stress values for all NMDS plots = 0.20593. (**a**) Samples from patients with PD are indicated in blue and healthy controls are indicated in red. The 95% confidence intervals around the centroids for each group are shown. Oral microbiomes in patients with PD are significantly different from healthy controls, as demonstrated by analysis of similarity (ANOSIM); R = 0.2372 and *p* < 0.001. (**b**) Identical NMDS plot, with samples color-coded by the abundance of bacteria from the phylum Bacteroidetes. (**c**) Identical NMDS plot, with samples color-coded by the abundance of bacteria from the phylum Proteobacteria. The strength and statistical significance were calculated based on a categorical variable found in the same mapping file. An R-value near 1 indicates dissimilarities between the groups, while 0 indicates no significant dissimilarities between the groups.

Analysis of beta-diversity is presented in Figure 2. Microbiota diversity was greater in healthy controls compared to patients with PD (Bray–Curtis F-value: 6.2; R-squared: 0.083558; *p*-value < 0.001; Jensen Shannon Divergence F-value: 10.032; R-squared: 0.12856; *p*-value < 0.001; Jaccard Index F-value: 4.3828; R-squared: 0.06055; *p*-value < 0.001). This is shown in Figure 3.

**Figure 4:**
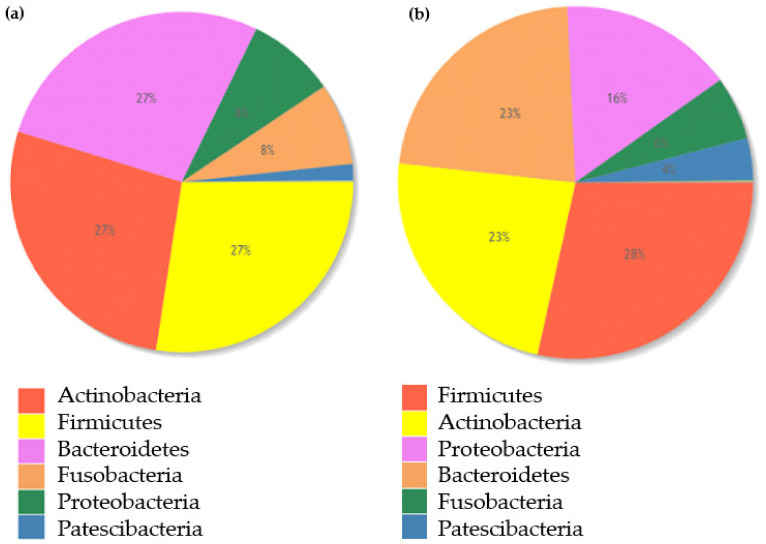
(**a**) Percentage distribution of the oral cavity microbiota of the patients with PD at the phylum level. (**b**) Percentage distribution of the microbiota of the oral cavity of the healthy controls at the phylum level.

Bacteroidetes (27%), Firmicutes (27%), and Actinobacteria (27%) constituted the central representation of bacteria in the oral cavity of patients with PD, while Firmicutes (28%), Proteobacteria (23%), and Actinobacteria (23%) occurred mainly in the healthy controls. The oral microbiota composition of the patients with PD and healthy controls are presented in Figure 4.

**Figure 5:**
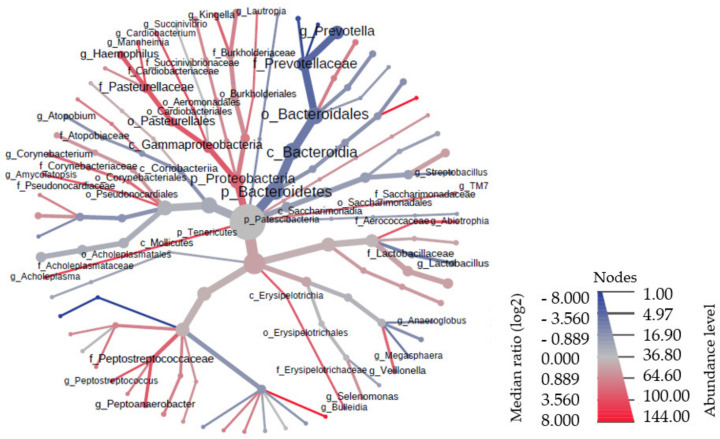
Phylogenetic tree of bacteria in the oral cavity of patients with PD.

The analysis of the two most common microbiome clusters (Proteobacteria and Bacteroidetes) showed a much higher number of Proteobacteria in the healthy controls (*p* < 0.000), while Bacteroidetes were significantly more abundant (*p* < 0.000) in the healthy controls. Moreover, a similar number of Proteobacteria were demonstrated in all patients with PD. Figure 5 presents the phylogenetic tree, taking into account the size of the individual taxonomic categories of the oral microbiome among patients with PD. The most numerous clusters were Bacteroidetes.

Oral microbiota in patients with PD included significantly more bacteria from genera *Prevotella*, *Streptobacillus*, *Megaspheaera*, and *Lactobacillus*. The healthy controls had oral microbiota more abundant in bacteria from genera *Haemophilus*, *TM7*, and *Veillonella*.

**Figure 6:**
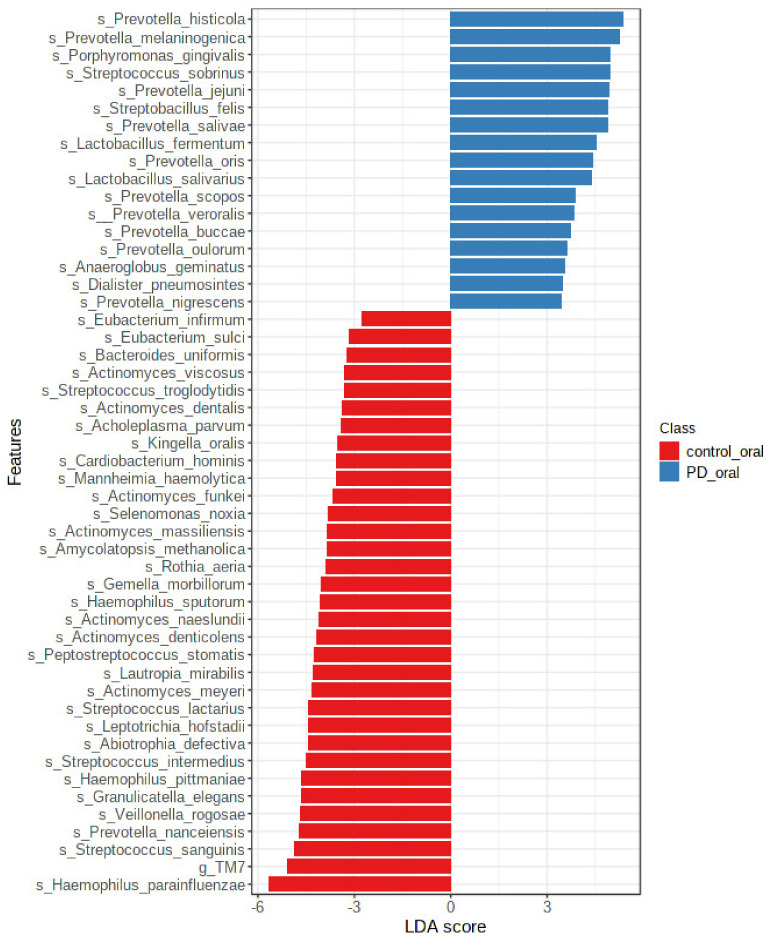
There are differences in oral microbiota between patients with PD and the healthy controls at the genre level.

The most numerous species in the patients with PD were *Prevotella histicola*, *Prevotella melaninogenica*, and *Porphyromonas gingivalis*. The healthy controls had oral microbiota more abundant in bacteria from species *Haemophilus parainfluenzae*, *Streptococcus sanguinis*, *Prevotella nanceiensis*, and other species presented in Figure 6.

### 3.4. Correlation of Microbiota with Food Preferences

Pearson correlation analysis revealed the species of bacteria which correlated with the frequency of some food products (Table 5). Several species of bacteria correlated positively with the frequency of consumption of several products. For example, the high abundance of *Veillonella rogosae* correlated with the high frequency of leaf vegetable consumption. The high abundance of *Prevotella histicola* correlated with a low frequency of red meat consumption. 

## 4. Discussion

Currently, microbiota (both intestinal and oral) is a popular subject in current research, and it seems to have a significant impact on multiple neurodegenerative diseases [27]. In the presented study, we attempted to correlate bacterial microbiota composition in the oral cavity of patients with PD and the influence of consumed components of the Western diet on the development of the disease. We decided to conduct this research to better understand a disorder which remains a significant challenge for modern medicine [28]. Additionally, our study was extended to compare patients with PD to healthy controls.

We used the 16S rRNA molecular technique to analyze oral microbiota, which allowed us to assess the diversity of microbiota and composition of microbiota present in a given environment by precisely sequencing the bacterial DNA. Additionally, we compared the dietary ingredients consumed by both groups to validate the presented outcomes. It is essential to notice that differences in microbiota composition can result from factors associated with diet. The oral cavity is the residency environment for more than 700 bacterial species. The microbiome is both hydrated and nourished by saliva. The pH 6.5 to 7.5 and the 37 °C temperature are conditions that enable pathogenic and mutualistic bacteria to coexist. In healthy individuals, the predominant oral microbial communities belong to Firmicutes (genus *Streptococcus*, family Veillonellaceae, genus *Granulicatella*), Proteobacteria (genera *Neisseria, Haemophilus*), Actinobacteria (genera *Corynebacterium*, *Rothia*, *Actinomyces*), Bacteroidetes (genera *Prevotella*, *Capnocytophaga*, and *Porhyromonas*), and Fusobacteria (genera *Fusobacterium*) [29]. The most numerous are Actinomyces, Capnocytophaga, Eikenella, Eubacteria, Fusobacterium, Haemophilus, Lactobacterium, Leptotrichia, Neisseria, Porphyromonas, Prevotella, Propionibacterium, Peptostreptococcus, Streptococcus, Staphylococcus, Veillonella, and Treponema [29,30]. Multiple factors such as tobacco and alcohol may interfere with the composition of the oropharynx microbiota and may lead to chronic inflammatory [31].

In this study, we showed statistically significant differences in the oral microbiome of patients with PD compared to healthy controls. The oral microbiome of patients with PD was characterized mainly by the high abundance of genera *Prevotellaceae, Lactobacillaceae*, *Streptobacillaceae*, and *Lactobacillaceae*, which are regular residents of the oral microflora. However, it has been reported that the genus *Prevotella* may also be pathogenic and prevalent in disease infections [32]. The increased abundance of Prevotella spp. Has been as a critical factor in developing persistent inflammation in the gut, causing mucosal dysfunction and systemic inflammation [32]. Systemic inflammation is related to the progression of different diseases, including PD.

Moreover, Streptococcus spp., which have also been increased in our study, were related previously to gastritis in patients without Helicobacter pylori infections [33]. This study also reported statistically different results related to food preferences between the patients with PD and the healthy controls. We noticed that, in patients with PD, the consumption of margarine, fish, red meat, avocado, olives, and cereal products was higher than in healthy controls. Deficient consumption was observed for thick groats, sweetened drinks, peanuts, dark bread, fruits, vegetables, and soy in the same group of patients with PD.

A study by Qu et al. found a consistent association between PUFA consumption and a lower risk of PD, while higher consumption of cholesterol and arachidonic acid was associated with an increased risk of PD, and our results showed no difference between the consumption of these fats in the two groups [34]. Our research did not show statistical significance in protein consumption between the two groups. This is in contrast to the results from the study of Honglei et al., which confirmed the association of high dairy consumption with an increased risk of PD behavior, especially in men [35]. In this study, increased consumption of animal fats in patients with PD was observed [34]. Our research has shown a low consumption of nuts, vegetables, and fruits in patients with PD, which is associated with a decrease in resveratrol. According to Arbo et al.’s research, this substance has a neuroprotective effect, including regulating pro-apoptotic proteins that affect cell death or counteracting changes in the morphology of mitochondria and the potential of the mitochondrial membrane [36,37,38,39]. The relationship between bacteria of the oral cavity and food preferences revealed the highest, positive correlation of *Veillonella rogosae* with the high frequency of leaf vegetable consumption. The highest negative correlation was observed for *Prevotella pallens* and alcohol consumption. Partially, our results differ in the diversity of bacteria compared to other studies, for example, results reported by Pedro A.B Pereira et al., which distinguish more types of bacteria in patients such as Streptococcus, Haemophilus, Neisseria, and Veillonella [16]. In this study, the most numerous were *Prevotella histicola, Prevotella melaninogenica*, and *Porphyromonas gingivalis.* In another study by Rozas et al. the most numerous were Lactobacillus, *Tannerella forsythia*, and *Prevotella intermedia* [18]. The differences between the bacterial composition may depend on genetic factors, the inhabited area, type of diet, illnesses, stimulants, stress, or medications used by patients. This study revealed the perturbation in oral microbiota of patients with PD, which differed from those of the healthy controls. The microbial biomarkers that we discovered may contribute to the pathogenesis of PD. The results that we demonstrate in this report may be a critical window in PD and microbiota development, creating the opportunity for novel therapies in the future. Unlike genetics, the microbiota, oral microbiota seems to be an easy target of modifications throughout life. Understanding the interaction between the oral cavity microbiota composition and PD progression is very important in nutritional-based interventions, especially in high-risk groups.

The size of the study was determined based on advanced technologies for microbiota composition analysis, which are associated with the high cost of such research. This study is associated with several limitations. The most critical was that the study group was not particularly abundant, and a more extensive study cohort would yield more precise results. There is also a significantly high percentage (79.7%) of people in the control group who have only primary or lower education, and this is essential to consider when evaluating their health literacy and priorities in choosing a diet. Moreover, the group of patients with PD included demented people, with a condition that could impact dental hygiene and oral microbiota composition.

Additionally, it is essential to note that the presented study is comparative, introducing potential bias. Therefore, it is essential to preserve caution when generalizing presented outcomes.

For researchers who would like to expand the research topic, we recommend including more diversified groups of patients, and they should present a broader spectrum of socioeconomic status and consider further education. Patients with PD should also be divided into more groups depending on the disease stage, which allows verification of whether the influence of the diet is similar for advanced PD patients. Moreover, we recommend analyzing the changes in the intestinal and nasal microbiome.

## 5. Conclusions

These findings suggest that oral microbiota could be associated with the development of PD. *Firmicutes* and *Actinobacteria* dominated the oral cavity microbiome in both examined groups. Patients with PD stand out with a higher concentration of phylum *Bacteroidetes,* and the healthy controls were recognizable because of the significant participation of *Proteobacteria*.

## Figures and Tables

**Table 1:** Characteristics of patients with PD and age-matched healthy controls.

	Group	N	M	SD	Min	Max	Q25	Me	Q75
Age (years)	PD	59	69.34	7.07	55.00	82.00	65.00	68.00	75.00
	Controls	108	64.21	10.23	51.00	82.00	54.50	62.00	71.50
Weight (kg)	PD	59	76.91	14.52	48.00	103.00	67.25	79.00	88.25
	Controls	108	72.95	13.38	47.00	118.00	65.00	70.00	80.00
Height (m)	PD	59	1.70	0.11	1.50	1.86	1.62	1.70	1.78
	Controls	108	1.68	0.08	1.50	1.90	1.64	1.66	1.75
BMI	PD	59	26.33	2.92	19.23	32.30	25.14	26.53	28.16
	Controls	108	25.81	3.75	18.36	33.22	22.92	25.56	28.17

N—numbers; M—mean; SD—standard deviation; Min—minimum; Max—maximum; Q—quartile; Me—median.

**Table 2:** The results of urine analysis in patients with PD.

Analysis	Result	Reference Values
Urine color	yellow/dark amber (24/3)	yellow
Clarity	clear/cloudy (22/5)	clear
Acidity	normal/acidic (17/10)	normal
Specific gravity	1020 ± 0.02	1020 ± 0.02
Glucose	negative	negative
Ketones	negative	negative
Nitrates	negative	negative
Bilirubin	negative	negative
Urobilinogen	negative	negative
Blood	≤3 red blood cells	≤3
Red blood cells	≤ 2 RBCs/hpf	≤2
White blood cells	≤2–5 WBCs/hpf	≤2–5
Protein	≤150 mg/day	≤150 mg/day
Squamous epithelial cells	negative	negative
Casts	negative	negative
Crystals	negative	negative
Bacteria	none/present (22/5)	none
Yeast	none	none

**Table 3:** The frequency of products consumed by patients with PD compared to the healthy controls. Table 3 shows the products with higher intake in patients with PD compared to healthy controls.

Product	Differences (%)	*p*-Value
Margarine	74.07	0.007
Fish	40.24	0.001
Breakfast cereal products	27.67	0.030
Avocado, olives	26.74	0.001
Red meat	26.04	0.015

**Table 4:** The frequency of products consumed by patients with PD compared to healthy controls. Significance testing was performed using the R statistical package. Table 4 shows the products with lower intake in the PD group compared to the controls.

Product	Differences * (%)	*p*-Value
Thick groats	−51.96	0.001
Sweetened drinks	−39.77	0.012
Dark bread	−36.89	0.001
Peanuts	−35.13	0.001
Juices	−31.14	0.004
Canned fruits	−23.31	0.055
Soy	−19.94	0.028
Dry fruits	−17.28	0.285
Fresh fruits	−14.58	0.008
Cross vegetables	−14.18	0.001

* Minus means deficiency.

**Table 5:** The Pearson’s correlation values show the relationship between bacteria and the frequency of consuming particular food products. Blue color means the most potent positive correlations, while red means the most potent negative correlations.

	Cross Vegetables	Yellow Vegetables	Leaf Vegetables	Parsley	Root Vegetables	Tomatoes	Chicken	Soy	Peanuts
Prevotella nanceiensis	−0.32	0.15	0.24	−0.23	0.14	0.02	−0.34	−0.03	−0.05
Haemophilus pittmaniae	−0.07	0.31	−0.03	−0.03	0.16	0.33	−0.12	−0.04	−0.26
Streptococcus sanguinis	−0.18	−0.20	−0.16	−0.10	−0.14	−0.24	0.23	0.03	0.15
Veillonella rogosae	0.21	0.28	0.41	0.04	0.23	0.25	−0.27	−0.12	−0.05
Haemophilus parainfluenzae	0.13	0.10	0.13	0.00	0.18	0.19	−0.08	0.03	0.09
Porphyromonas gingivalis	−0.04	−0.05	0.08	−0.08	0.21	0.02	0.18	−0.20	−0.24
Streptobacillus felis	−0.34	−0.04	0.06	−0.10	0.13	−0.10	0.04	−0.24	−0.13
Prevotella salivae	−0.10	−0.22	0.03	0.19	0.13	−0.07	0.11	−0.03	0.05
Prevotella pallens	−0.12	−0.07	0.10	−0.02	0.17	−0.19	−0.14	−0.03	0.19
Megasphaera micronuciformis	0.10	0.20	0.22	0.21	0.30	−0.01	−0.05	0.08	0.06
Prevotella jejuni	−0.05	−0.30	0.23	0.28	0.18	−0.23	0.08	0.09	0.32
Prevotella histicola	0.05	−0.02	0.25	0.16	0.04	0.00	−0.13	0.20	0.02
Prevotella melaninogenica	−0.29	−0.52	0.00	−0.09	0.01	−0.13	−0.09	−0.16	0.20
Streptococcus sobrinus	0.01	0.22	0.06	−0.09	−0.13	−0.07	−0.27	−0.10	−0.10
	**Fruit**	**Apricots**	**Avocado and Olives**	**Dry Fruits**	**Fresh Fruits**	**Canned Fruits**	**Milk**	**Eggs**	**Dark Bread**
Prevotella nanceiensis	0.07	−0.23	0.03	−0.04	−0.08	0.07	−0.20	−0.40	−0.13
Haemophilus pittmaniae	0.19	−0.31	−0.04	−0.07	−0.01	0.12	0.13	−0.11	−0.20
Streptococcus sanguinis	−0.04	−0.08	−0.10	0.14	−0.13	−0.16	0.38	−0.02	0.03
Veillonella rogosae	0.19	−0.20	0.02	−0.08	0.14	−0.06	0.17	0.14	0.13
Haemophilus parainfluenzae	0.17	−0.02	0.07	0.09	0.37	−0.03	0.19	0.06	0.09
Porphyromonas gingivalis	−0.53	−0.34	−0.25	−0.22	−0.21	−0.13	−0.35	−0.12	−0.28
Streptobacillus felis	0.06	0.08	−0.04	−0.14	0.14	−0.14	−0.16	−0.12	−0.26
Prevotella salivae	0.05	0.26	0.08	0.09	0.26	0.31	0.02	−0.28	−0.16
Prevotella pallens	−0.04	0.03	0.19	0.10	−0.08	0.15	−0.08	−0.43	−0.05
Megasphaera micronuciformis	0.00	0.26	0.24	0.06	0.04	0.06	−0.02	−0.45	−0.03
Prevotella jejuni	0.09	0.09	0.21	0.29	−0.03	0.13	0.19	−0.22	0.10
Prevotella histicola	0.05	0.38	0.01	0.13	0.38	0.18	−0.18	−0.25	0.04
Prevotella melaninogenica	0.17	0.03	−0.07	0.16	0.22	0.23	0.01	−0.04	−0.10
Streptococcus sobrinus	−0.26	0.03	0.11	−0.21	−0.36	−0.42	−0.10	−0.07	0.26
	**White Bread**	**Thick Groats**	**Breakfast Cereal Products**	**Butter**	**Margarine**	**Plant Oils**	**Red Meat**	**White Meat**	**Fish**
Prevotella nanceiensis	0.10	−0.20	0.25	−0.17	0.15	−0.22	−0.28	−0.18	−0.15
Haemophilus pittmaniae	0.18	−0.19	0.34	0.04	−0.02	0.02	0.05	−0.31	−0.14
Streptococcus sanguinis	0.01	−0.10	−0.17	0.09	−0.06	0.35	−0.03	0.12	0.06
Veillonella rogosae	0.04	−0.16	0.19	0.12	−0.13	−0.31	0.07	−0.11	−0.12
Haemophilus parainfluenzae	−0.02	0.03	0.22	−0.04	−0.01	−0.24	−0.18	−0.20	−0.09
Porphyromonas gingivalis	0.21	−0.12	0.16	0.16	−0.19	0.19	0.20	−0.25	−0.12
Streptobacillus felis	−0.26	−0.20	−0.11	−0.35	0.31	−0.23	−0.33	−0.03	−0.16
Prevotella salivae	0.00	0.07	0.02	−0.04	−0.02	−0.18	−0.26	−0.19	0.00
Prevotella pallens	0.13	−0.16	0.00	−0.09	0.06	−0.22	−0.15	−0.02	−0.03
Megasphaera micronuciformis	−0.07	0.16	0.01	−0.17	0.10	−0.11	−0.34	−0.25	−0.05
Prevotella jejuni	−0.03	−0.12	0.07	0.13	−0.18	−0.23	−0.20	−0.15	−0.19
Prevotella histicola	−0.17	0.33	0.02	−0.32	0.27	−0.08	−0.44	−0.13	−0.17
Prevotella melaninogenica	0.13	−0.22	0.06	0.26	−0.28	0.08	0.18	−0.14	−0.18
Streptococcus sobrinus	−0.08	−0.10	−0.25	−0.28	0.31	−0.07	0.07	0.10	−0.06
	**Juices**	**Sweetened Drinks**	**Coffee**	**Alcohol**	**Amount of Water**	**Frying**	**Cooking**	**Baking**	**Suffocation**
Prevotella nanceiensis	−0.19	0.03	−0.47	−0.26	−0.06	−0.36	0.03	−0.20	0.04
Haemophilus pittmaniae	0.05	0.30	−0.13	0.03	−0.17	−0.12	0.00	−0.46	−0.19
Streptococcus sanguinis	−0.05	0.12	0.04	−0.16	−0.02	0.17	−0.08	0.08	−0.14
Veillonella rogosae	0.07	−0.01	0.06	−0.04	−0.10	0.04	0.00	−0.17	−0.31
Haemophilus parainfluenzae	−0.21	−0.18	−0.30	−0.21	−0.03	−0.37	0.03	−0.51	−0.65
Porphyromonas gingivalis	0.16	−0.02	0.09	0.01	−0.24	0.17	0.02	−0.09	0.11
Streptobacillus felis	0.00	−0.22	−0.28	−0.13	0.03	−0.17	−0.29	0.15	0.06
Prevotella salivae	0.03	−0.09	−0.29	−0.24	−0.33	−0.27	−0.32	0.00	0.11
Prevotella pallens	−0.20	−0.10	−0.49	−0.48	−0.18	−0.22	−0.05	−0.04	0.30
Megasphaera micronuciformis	−0.09	−0.10	−0.37	−0.42	−0.04	−0.33	−0.19	0.03	0.16
Prevotella jejuni	−0.17	−0.17	−0.29	−0.36	−0.30	−0.12	−0.32	−0.04	0.01
Prevotella histicola	0.02	−0.21	−0.23	−0.25	−0.03	−0.31	−0.14	0.02	0.02
Prevotella melaninogenica	−0.26	−0.01	−0.03	−0.12	−0.52	0.16	−0.01	−0.04	−0.14
Streptococcus sobrinus	−0.17	−0.07	0.10	0.16	0.36	−0.11	0.00	−0.01	0.09
